# Proof of Principle for a Novel Class of Antihypertensives That Target the Oxidative Activation of PKG Iα (Protein Kinase G Iα)

**DOI:** 10.1161/HYPERTENSIONAHA.117.09670

**Published:** 2017-08-09

**Authors:** Joseph R. Burgoyne, Oleksandra Prysyazhna, Daniel A. Richards, Philip Eaton

**Affiliations:** From the Cardiovascular Division, the British Heart Foundation Centre of Excellence, the Rayne Institute, St Thomas’ Hospital, King’s College London, United Kingdom.

**Keywords:** blood pressure, hypertension, risk factor, therapeutics, vasodilation

## Abstract

Arterial hypertension continues to be a major health burden. Development of new antihypertensive drugs that engage vasodilatory mechanisms not harnessed by available therapies offer therapeutic potential. Oxidants induce an interprotein disulfide in PKG Iα (protein kinase G Iα) at C42, which is associated with its targeting and activation, resulting in vasodilation and blood pressure lowering. Consequently, we developed an assay and screened for electrophilic drugs that activate PKG Iα by selectively targeting C42, as such compounds have potential as novel antihypertensives with a mechanism of action that differs from current therapies. In this way, a drug that we termed G1 was identified, which targets C42 of PKG Iα to induce vasodilation of isolated resistance blood vessels and blood pressure lowering in a mouse model of angiotensin II–induced hypertension. In contrast, these antihypertensive effects were deficient in angiotensin II–induced hypertensive C42S PKG Iα knockin mice. These transgenic mice were engineered to have the reactive cysteinyl thiol replaced with a hydroxyl so that it cannot react with endogenous vasodilatory oxidants or electrophiles such as drug G1. These studies, therefore, provide validation of PKG Iα C42 as the target of G1, as well as proof-of-principle for a new class of antihypertensive drugs that have potential for further development for clinical use in humans.

## Abstract 2

Supplemental Digital Content is available in the text.

**See Editorial Commentary, pp 490–492**

Arterial hypertension is a common, albeit modifiable risk factor, for cardiovascular disease and mortality. Fortunately, there are several classes of antihypertensive therapies that alone or in combination are effective in lowering blood pressure. Reducing blood pressure limits organ damage and adverse cardiovascular disease outcomes. Despite this antihypertensive armoury, new drugs that engage mechanisms of actions not harnessed by current compounds could provide valuable alternate first-line or complementary therapies to improve the treatment of high blood pressure. Such compounds that operate by a different mode of action also offer the theoretical prospect of treating the significant number of patients who are resistant to current therapies,^1–4^ which represents a significant unmet clinical need.

Because obesity, diabetes mellitus, and increased age are major risk factors for hypertension and because the population is living longer and becoming increasingly overweight, additional pharmacotherapies may prove valuable in treating the high blood pressure pandemic. Consistent with this need to improve the treatment of hypertension, pharmaceutical companies and academic researchers have programs to develop novel blood pressure–lowering drugs or interventional approaches, some involving devices, that can achieve this.^5^

PKG (protein kinase G) is well established as the end-effector kinase in blood vessel dilation, facilitating blood pressure lowering in response to agents that elevate NO. NO binds soluble guanylate cyclase, activating it to generate cyclic GMP that bind PKG to induce allosteric activation and then phosphorylation of many end-effector proteins that mediate vasodilation. Oxidant-induced disulfide formation in PKG Iα is an alternate mechanism by which this kinase can be activated and contributes, at least in part, to the endothelium-derived hyperpolarizing factor (EDHF) mechanism of vasodilation and blood pressure lowering. EDHF predominates over NO-cGMP– or prostacyclin-dependent mechanisms of vasodilation in resistance blood vessel control of blood pressure,^6–8^ operating in various vascular beds in many species, including humans.^9–11^

Current blood pressure–lowering drugs do not use this mechanism. Because this oxidant-induced activation is a major mechanism of blood pressure lowering as described above, drugs that may recruit this pathway are anticipated to be effective vasodilators. As such, a compound, which would represent a unique drug class, stimulating a major endogenous mechanism responsible for blood pressure lowering in vivo, this may provide additional therapeutic strategies in addition to current treatment options. Furthermore, it is rational that this new class of drug may in theory perhaps also treat hypertension that is resistant to current therapies, for which there is a significant unmet clinical need.

With the considerations above in mind, we set out to identify drugs that recruit the oxidative activation of PKG Iα. Our strategy of recruiting a major component of the blood pressure–lowering EDHF mechanism, by specifically targeting C42, which is unique to PKG Iα, is a rational approach that was anticipated to potentially yield a selective and highly effective drug. Our vision was that a drug capable of inducing or mimicking the interprotein disulfide in PKG Iα would selectively react with C42 to target and activate the kinase, thus facilitate blood pressure lowering. Thus, we screened a library of electrophilic compounds, assessing their ability to induce oxidation of recombinant PKG Iα.

To do this, we developed an assay using recombinant PKG Iα and dibromobimane (dBBr), which fluoresces when it adducts vicinal thiols. Because the C42 residues on the adjacent parallel-aligned chains of PKG Iα are vicinal, when dBBr is added to the kinase in the reduced state, it fluoresces (Figure 1A). However, if a drug induces oxidation of C42, this will attenuate the adduction of dBBr with the kinase and so reduce the fluorescence compared with vehicle-treated control (Figure 1A). Thus, if pretreatment of the kinase with a drug attenuates the fluorescence signal obtained when PKG Iα and dBBr are mixed, this would be consistent with C42 oxidation and therefore further investigated. Having successfully identified such compounds, we then assessed their ability to induce vasodilation of isolated wild-type mesenteric arteries, with those that did so effectively undergoing a subsequent counterscreen. This counterscreen involved repeating the assessment of such compound to dilate the WT mesenteric preparation but concomitantly also assessing responses in mesenteries from C42S PKG Iα knockin (KI) mice. In this way, we identified a compound, named G1, which efficiently relaxes WT but not KI vessels, which was then assessed in a murine model of hypertension. G1 lowered blood pressure in hypertensive WT, but not KI, mice in vivo. This provides proof of concept that drugs that activate PKG Iα by targeting C42 are a realistic strategy for generating novel antihypertensive medicines.

**Figure 1.**
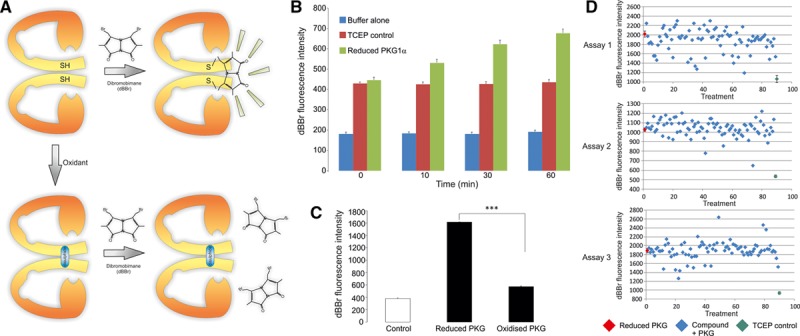
Overview of dibromobimane screening method and its validation. **A**, Schematic of the method used to identify drugs that target C42 of PKG Iα (protein kinase G Iα). As C42 on each of the chains of the kinase are vicinal to each other, this enables the bifunctional dibromobimane molecule to react with each residue, which results in it becoming fluorescent. Thus, dibromobimane provides readout of PKG Iα in the reduced state, and drugs that target C42 are anticipated to interfere with this and so lower the fluorescence signal compared with control. **B**, Validation studies showing combining dibromobimane with PKG Iα reduced with TCEP generates a time-dependent increase above control preparations. **C**, Quantification of dibromobimane fluorescence signal generated by reduced versus oxidized PKG Iα. **D**, Screening and identification of compounds that may target PKG Iα C42 using a 96-well plate fluorimetric assay identified several compounds that markedly lowered the dibromobimane-dependent fluorescence signal compared with control. Several of these, as shown in Table, were subsequently tested for their ability to dilate mesenteric blood vessels.

## Methods

### In Vitro Screen for Compounds That Induce Oxidation of PKG Iα

Molecules with potential electrophilic properties were obtained from InterBioScreen (http://www.interbioscreen.com/) and are individually listed in Table S1 in the online-only Data Supplement. In all assays, 1.1 µg/µL of recombinant PKG Iα (14–688; Merck Millipore) was reduced for 20 minutes at room temperature with 5 mmol/L TCEP. Reduced PKG Iα was then diluted to ≈1 µmol/L, based on the molecular weight of a monomer, in 100 mmol/L Tris-HCl pH 7.4 and 750 ng placed into wells of a 96-well plate. In initial experiments, the fluorescence of dBBr (100 µmol/L) was compared between reduced PKG Iα and TCEP-only controls over a 1-hour period. In further experiments, dBBr fluorescence was compared between reduced and air oxidised PKG Iα (20 minutes at room temperature without TCEP) 1 hour after the addition of 100 µmol/L dBBr. For the in vitro screen, 750 ng of reduced PKG Iα was placed into each well of a 96-well plate preloaded with 100 µmol/L drug/well. After 10-minute incubation at room temperature, 100 µmol/L dBBr was added to each well. After a further 60-minute incubation at room temperature, dBBr fluorescence (λex 393 nm; λem 477 nm) in each well was assessed using a microplate reader (SpectraMax GeminiXS; Molecular Devices).

### Animal Studies

All procedures were performed in accordance with the Home Office Guidance on the Operation of the Animals (Scientific Procedures) Act 1986 in UK and were approved by an institutional review committee. Mice constitutively expressing PKG Iα Cys42Ser were generated on a pure C57BL/6 background by Taconic Artemis as described before.^12^ Age-matched and body weight–matched WT or PKG Iα Cys42Ser KI male mice were used in all studies. All animals had ad libitum access to standard chow and water. Mice were kept under specific pathogen-free conditions and under a 12-hour day/night cycle.

### Myography

Second-order mesenteric arteries from WT or C42S PKG Iα KI mice were mounted in a Danish Myo Technology tension myograph, stretched to the optimal pretension condition with Danish Myo Technology normalization module and bathed in Krebs solution at 37°C with a 95% O_2_:5% CO_2_ environment. Vasotone measurements were made after wake up with KCl (60 mmol/L) by determining the responses of U46619-contracted (0.1 μmol/L) mesenteric vessels to cumulatively increasing concentrations of test compounds. In some studies, vascular rings were isolated from the thoracic aorta; carotid, renal (second order), or femoral arteries were also studied.

### Blood Pressure Measurements

Blood pressure and heart rate were assessed by radio telemetry in conscious freely moving mice as described before.^12^ Alzet osmotic mini-pumps were used to deliver angiotensin II at 1.1 mg/kg per day in some studies. Drug G1 was delivered intraperitoneally (3.7–14.8 mg/kg) or orally (20 mg/kg) in some studies. To deliver G1 orally, without stress or risk of dislodging the telemetric probe catheter, it was provided suspended in water and set in gelatin flavored with sodium saccharin.

### Assessing Vasodilator-Stimulated Phosphoprotein Phosphorylation

Rat aortic smooth muscle cells were maintained in Dulbecco modified eagle medium (GIBCO, Life Technologies) supplemented with 10% fetal calf serum and 1% penicillin/streptomycin and kept at 37°C in an incubator with 5% CO_2_. To assess VASP (vasodilator-stimulated phosphoprotein) phosphorylation, smooth muscle cells grown on cell culture plates were exposed to varying concentrations of G1, 8-Br-cGMP (Sigma) or a combination of both. After 10-minute incubation at 37°C, cells were lysed into sample buffer and then assessed for VASP Ser239 phosphorylation using Western immunoblotting (Cell Signaling).

### Monitoring PKG Iα Disulfide Dimerization

Western immunoblotting was used to determine the redox state of PKG Iα as described previously,^12^ with maleimide (100 mmol/L) used in preparation buffers to alkylate thiols and prevent thiol disulfide exchange. Antibody ADIKAP-PK005 (Enzo Life Science) was used to probe blots for PKG Iα. Horseradish peroxidase–linked secondary antibody (Cell Signaling) and ECL reagent (GE Healthcare) were used. Digitized immunoblots were analyzed quantitatively with a Gel-Pro Analyzer 3.1. The amount of PKG Iα disulfide dimer in each sample was indexed by expressing the immunoblot signal at the dimeric weight as a percentage of the combined monomeric and dimeric signals.

### Statistics

Differences between groups were assessed using ANOVA where appropriate, followed by Student *t* test when only 2 groups were tested or a Tukey test when ≥3 groups were compared. Differences were considered significant at the 95% confidence level (*P*<0.05).

## Results

Figure 1A provides a schematic overview of the principle of the dBBr-screening assay that was used in combination with recombinant PKG Iα to identify candidate compounds that may induce oxidation of the kinase. C42 on adjacent chains of the PKG Iα homodimer is within a few Angstroms of each other^13^ and susceptible to oxidative disulfide conjugation.^14^ dBBr is capable of reacting with each of the 2 C42 residues within the homodimer complex because of their proximity, with cross-linking inducing florescence. In perhaps the simplest interpretation of this assay system, a drug that induced a disulfide between the kinase subunits would then prevent generation of a dBBr-dependent fluorescent signal as the requisite C42 thiols would no longer be available. Figure 1B shows that an assay mixture that contained recombinant PKG Iα and TCEP, which is included to maintain the kinase in the reduced state, generates a time-dependent signal over 60 minutes. When PKG Iα was selectively removed, dBBr failed to generate a signal above baseline, indicating that the signal generated was dependent on the presence of the kinase. Overall, this indicates that the dBBr assay was suitable for assaying the oxidation state of PKG Iα. Consistent with this, when PKG Iα in the reduced state was compared with oxidised kinase, there was a clear and reproducible difference in dBBr-dependent fluorescence observed at the 60-minute time point (Figure 1B).

Next, the compound library was tested in the dBBr assay system, which being in a 96-well plate format allowed the efficient and rapid screening of ≈300 candidate compounds. The signal generated by reduced PKG Iα, because its C42 thiol groups are available to react efficiently with dBBr, is shown in red. Compounds of particular interest were those that substantively attenuated dBBr-dependent fluorescence, as this was likely because they induced oxidation of C42. On this basis, 12 compounds, whose chemical structures are shown in Table, were chosen for further study–as is the extent to which they prevented dBBr interaction with PKG Iα.

**Table.**
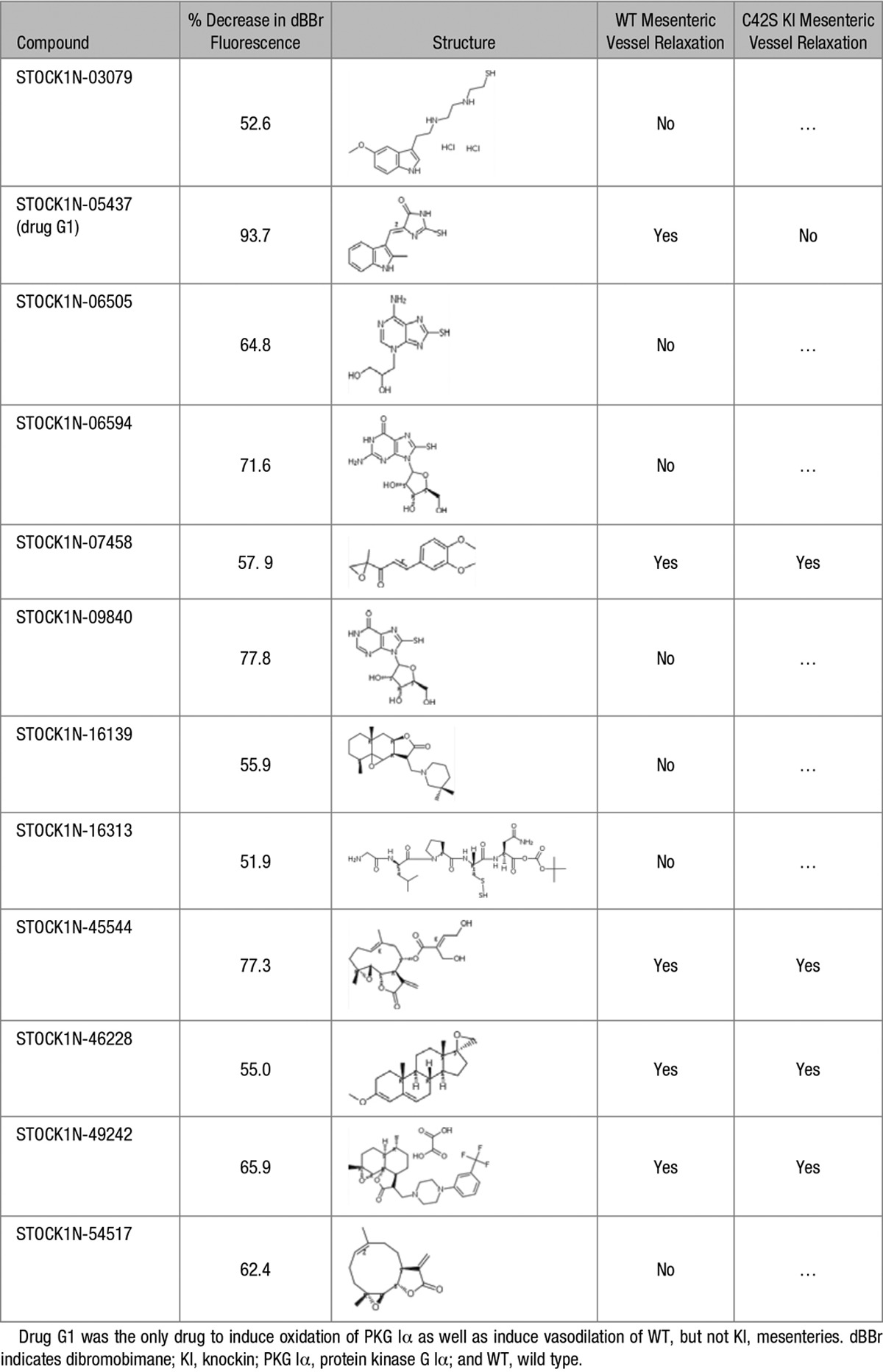
Summary of Molecules Studied in Detail in This Study

**Figure 2.**
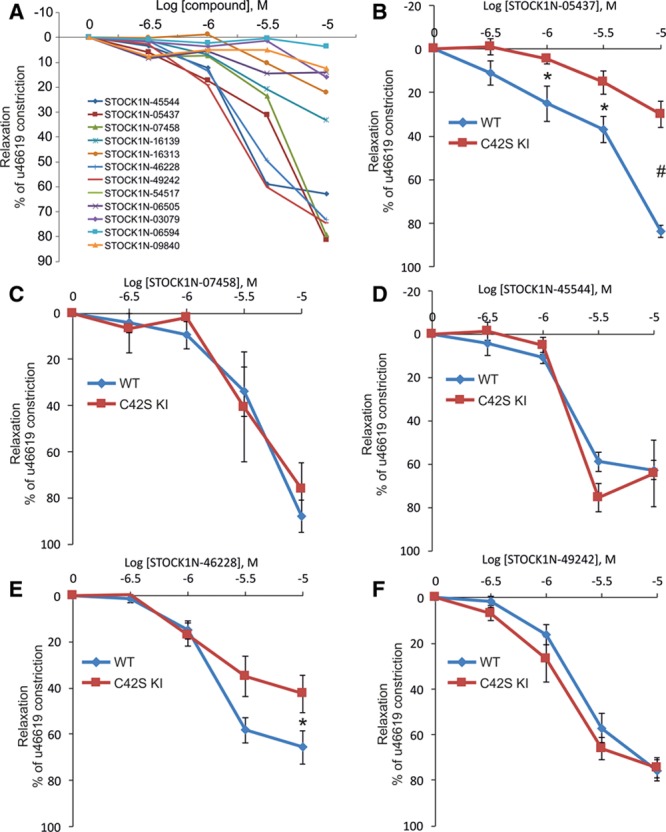
Screening candidate compounds for their ability to dilate mesenteric vessels by specifically targeting PKG Iα (protein kinase G Iα) C42. **A**, Twelve compounds that the fluorescence screen assay identified as likely targeting PKG Iα C42 were examined for their ability to dilate mesenteric arteries from wild-type mice, which 5 of them did efficiently. **B**–**F**, The 5 compounds that induced efficient vasorelaxation were assessed again, repeating the assessment of their ability to dilate vessels from wild-type mice, but at the same time comparing their ability to do this in mesenteries from C42S PKG Iα KI mice. As summarized in Table, a compound that we have called G1 efficiently dilated vessels from wild-type but not those from the knockin mice. Drug G1 was considered a hit compound that we then assessed for its ability to lower blood pressure in vivo.

The 12 compounds were examined for their ability to relax isolated WT mesenteric artery preparations using wire myography (Figure 1D). In this way, 5 compounds were identified for follow-up myography studies, repeating the assessment of their ability to relax WT mesenteries but concomitantly also investigating their comparative abilities to dilate vessels from the C42S PKG Iα KI mice (Figure 2B through 2F). All 5 compounds again relaxed the WT preparations, so corroborating the previous observations, but only 1 of the compounds failed to induce efficient relaxation in mesenteries from the C42S PKG Iα KI (Table). Thus, compound STOCK1N-05437, which we subsequently refer to as drug G1, is effective at inducing vasodilation in WT but not KI preparations—consistent with it activating PKG Iα by targeting C42.

**Figure 3.**
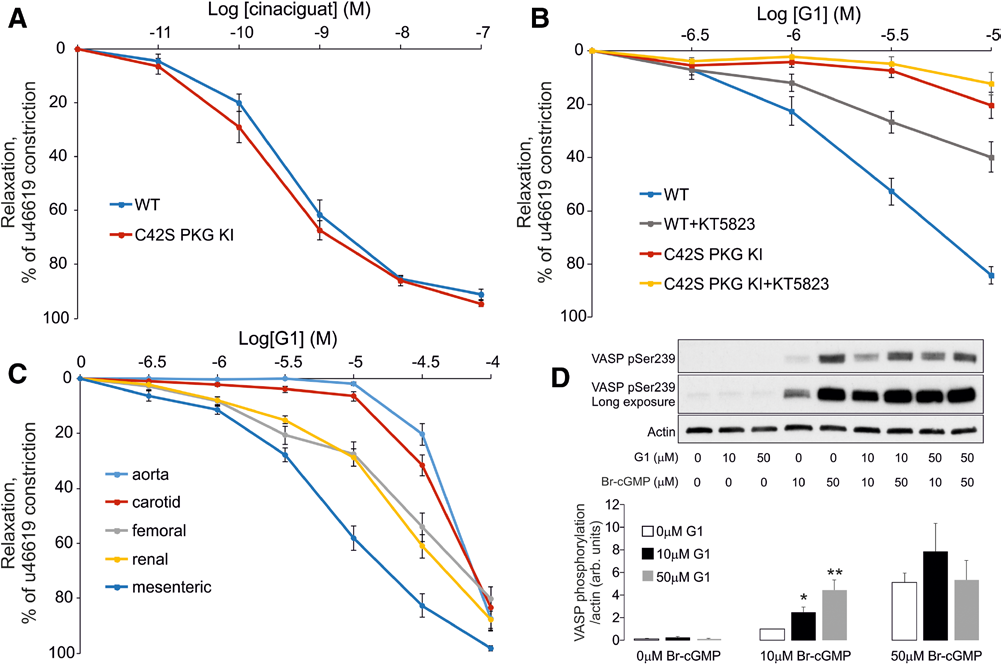
Drug G1-induced vasodilation involves cGMP-independent activation of PKG Iα (protein kinase G Iα) **A**, Cinaciguat induced comparable dose-dependent vasodilation in mesenteries isolated from wild-type (WT) or C42S PKG Iα knockin (KI) mice. **B**, G1-dependent vasodilation of mesenteries from WT was attenuated by the PKG inhibitor KT5823, as was the partial relaxation caused by the compound in vessels from KI mice. **C**, G1 caused vasodilation in arteries isolated from several vascular beds, inducing more potent vasodilatory actions in the smaller vessels. **D**, 10 or 50 mmol/L 8-Br-cGMP induced phosphorylation of VASP (vasodilator-stimulated phosphoprotein) S239 in smooth muscle cells, whereas G1 did not. When 10 or 50 mmol/L G1 and 10 mmol/L 8-Br-cGMP were concomitantly administered to the cells, there was significant potentiation of the VASP phosphorylation signal generated compared with 10 mmol/L 8-Br-cGMP alone.

Cinaciguat, which elevates cGMP to dilate blood vessels, dose dependently relaxed mesenteries from WT or KI to the same extent (Figure 3A). This comparable vasodilation to an agent that elevates cGMP, which is consistent with previous findings,^12^ contrasts the disparate responses of the 2 genotypes to G1. Vessels from KI mice are significantly deficient in their vasodilatory responses to G1 (Figure 3B), showing the importance of C42 in the mechanism of action of the compound. The PKG inhibitor KT5823 attenuated G1-dependent vasodilation in vessels from WT mice but also reduced the already impaired relaxation to the drug in mesenteries isolated from KIs (Figure 3B). G1 was also able to dilate vascular rings isolated from the thoracic aorta, as well as carotid, renal, and femoral arteries (Figure 3C).

The membrane-permeable cGMP mimetic 8-Br-cGMP, when applied to smooth muscle cells at 10 or 50 µmol/L, induced a concentration-dependent increase in VASP phosphorylation. In contrast, when cells were exposed to G1 alone at 10 or 50 mmol/L, this did not alter VASP phosphorylation (Figure 3D). However, it was notable that when cells were concomitantly exposed to 10 mmol/L 8-Br-cGMP and G1, there was a synergistic effect, with phosphorylation of VASP being greater than that with 10 µmol/L 8-Br-cGMP alone. When 8-Br-cGMP was applied at 50 mmol/L, the increase in phospho-VASP was robust, perhaps representing maximal phosphorylation, which would explain why cotreatment with G1 did not potentiate the phosphorylation signal.

**Figure 4.**
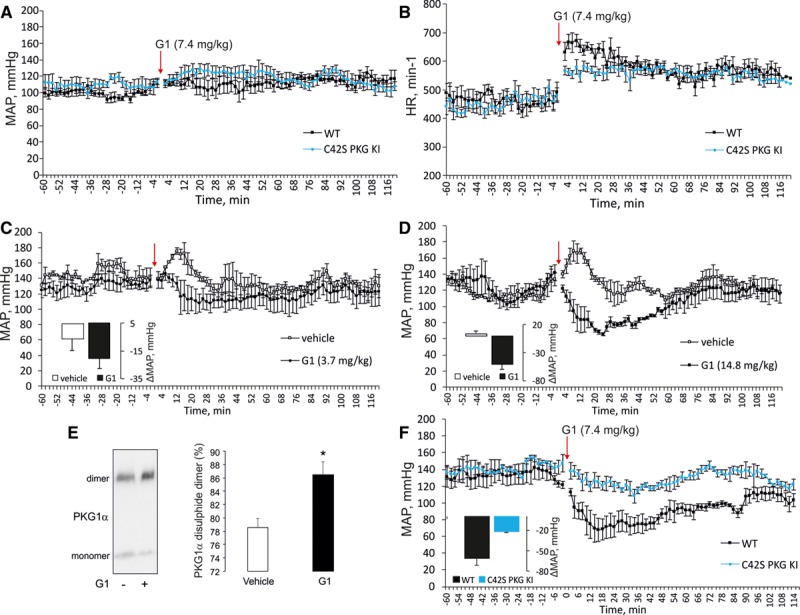
Drug G1 lowers blood pressure in hypertensive but not normotensive wild-type (WT) mice. **A** and **B**, G1 administered at 7.4 mg/kg to healthy WT mice did not alter their blood pressure but induces reflex tachycardia to increase heart rate. **C** and **D**, Mice were administered angiotensin II to increase their blood pressure, after which they were treated with 3.7 or 14.8 mg/kg G1 intraperitoneally, which decreased their blood pressure by 20.6±6.9 or 50.6±9.1 mm Hg, respectively. **E**, Aorta was isolated from mice exposed to angiotensin II and vehicle or angiotensin II and G1 and assessed for the redox state of PKG Iα (protein kinase G Iα). This showed that G1-induced oxidation of PKG Iα to the disulfide state in vivo. **F**, G1 efficiently lowered blood pressure of angiotensin II–induced hypertensive WT, but not C42S PKG Iα knockin (KI), mice.

G1 was next tested in vivo in healthy mice implanted with telemetric devices that allow blood pressure and heart rate to be constantly monitored. G1 or vehicle control was administrated by intraperitoneal injection, and the acute impact on hemodynamics assessed. Drug G1 administered at 7.4 mg/kg did not decrease blood pressure, but there was a concomitant reflex tachycardia (Figure 4A and 4B). When this was repeated using 14.8 mg/kg dose of G1, again blood pressure was not altered—but this higher dose induced a potentiated increase in heart rate (not shown). These reflex tachycardia responses are anticipated in response to a drug that induces vasodilation.^15^ As the observations presented above relating to G1-induced tachycardia were promising, WT or KI mice were administered angiotensin II for 7 days using an osmotic mini-pump to induce hypertension. On day 8 of this hypertension protocol, G1 (3.7 or 14.8 mg/kg) or vehicle was coadministered intraperitoneally. G1 induced a rapid, dose-dependent drop in mean arterial pressure in WT mice, which with the higher dose slowly recovered to basal during the ensuing ≈90 minutes (Figure 4C and 4D). Aorta was isolated from mice exposed to angiotensin II and vehicle or 14.8 mg/kg G1 for ≈20 minutes and assessed for the redox state of PKG Iα using Western immunoblotting, which showed that the drug had induced oxidation of the kinase to the interprotein disulfide state (Figure 4E). Intraperitoneally administered G1 was tested again using the angiotensin II hypertension model, but this time comparing the responses of WT to C42S PKG Iα KI mice. G1 efficiently lowered blood pressure in WT, but only partially in the KI (Figure 4F). However, it should be considered that in these initial experiments, the drug was administered intraperitoneally at a relatively high dose and so is anticipated to be more bioavailable than when the drug is provided orally, which was our ultimate goal.

**Figure 5.**
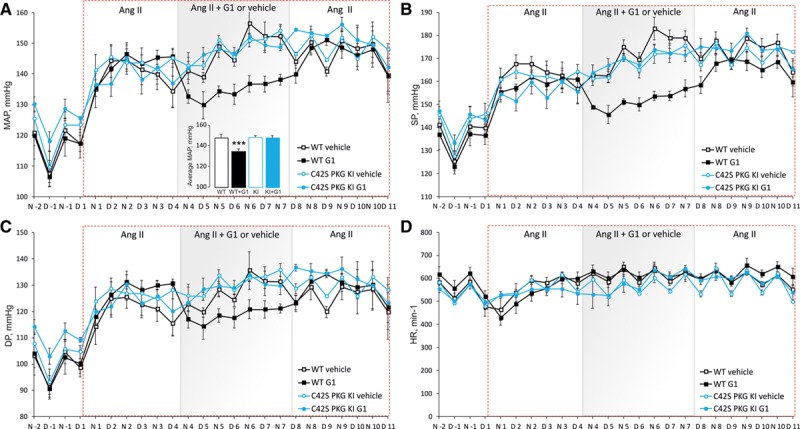
Oral drug G1 functions as an effective antihypertensive by targeting C42 of PKG Iα (protein kinase G Iα). **A**–**D**, Hypertension was induced in wild-type (WT) or knockin (KI) mice with angiotensin II. Each genotype was then administered vehicle or G1 (20 mg/kg per day) orally for 4 d in the continued presence of angiotensin II. It was evident that orally supplied G1, but not vehicle, was effective at lowering mean arterial pressure (MAP), systolic pressure (SP), and end-diastolic pressure (DP) in WT, but not KI, mice. Heart rate did not differ between genotype or drug treatment groups.

In subsequent studies using the same angiotensin II–induced hypertension model, G1 was next administered orally at 20 mg/kg for 4 days after which it was removed with continued hemodynamic monitoring. Orally administered G1 effectively lowered blood pressure in WT, whereas there was no blood pressure–lowering response in the KI or the treatment groups administered vehicle (Figure 5A through 5C). G1 had no impact on the heart rate compared with vehicle in either genotype. When G1 was removed, the blood pressure of the WT increased back to match those in the other 3 experimental treatment groups, providing further reassurance that G1 shows characteristics that would be anticipated for an antihypertensive. It may appear that G1 demonstrates tachyphylaxis (Figure 5C), as mean arterial progressively increased during the 4 days when the drug was administered. However, it is important to note that G1 is coadministered with angiotensin II, which when given alone continued to increase blood pressure. In fact, the delta decrease in mean arterial pressure achieved by G1 in the presence of angiotensin II compared with the angiotensin II plus vehicle group was 8.5 mm Hg on day 1, 14.6 mm Hg on day 2, 19.6 mm Hg on day 3, and 14.1 mm Hg on day 4.

## Discussion

There are many current pharmacotherapies that are reasonably efficacious in the treatment of systemic arterial hypertension. These include renin–angiotensin–aldosterone system antagonists, diuretics, and β-blockers, which alone or in combination are effective in lowering blood pressure. However, despite the availability of these antihypertensive drugs, the development of new pharmacotherapies with mechanisms of action that differ from current compounds may prove valuable in the treatment of high blood pressure. Such compounds may provide first-line therapies that complement those already used, but because of the disparate mode of action theoretically may also treat some patients with resistance to current therapies, for which there is an unmet clinical need.^1–4^

We identified a new mechanism by which PKG Iα can be activated by disulfide formation to relax blood vessels,^14,16,17^ which is important in vivo.^12^ We hypothesized that drugs that bind PKG Iα to induce or mimic the disulfide may have therapeutic blood pressure–lowering actions. Because disulfide activation of PKG Iα contributes to an endogenous mechanism of vasodilation,^12,17^ a drug that engages this mechanism may be especially efficacious in the context of blood pressure lowering. Furthermore, because the therapies that are currently used likely do not engage this disulfide-induced activation of PKG Iα mechanism of vasodilation, such compounds conceivably may work in scenarios where current therapies fail, such as resistant hypertension, as well as potentially providing an alternative or complementary approach to current treatments. Although nitroglycerin-dependent blood pressure lowering is partly mediated by oxidation of PKG Iα,^18^ this mechanism is likely a minor component as this drug also releases the potent vasodilator NO. Indeed, NO derived from nitroglycerin increases cGMP that binds to PKG Iα and limits its oxidation,^16,19^ further limiting the disulfide-mediated activation mechanism. Apart from this, sustained administration of nitroglycerin results in the tolerance phenomenon,^20^ whereby the drug stops working and so precludes its use for the chronic treatment of arterial hypertension. We only tested G1 for 4 days, during which it continued to lower blood pressure in the angiotensin II–induced hypertension model. It will be important in subsequent studies evaluating G1 that longer therapy durations be examined to more fully rule out the possibility of tachyphylaxis as occurs with the nitroglycerin tolerance. Although it should be noted that when we studied nitroglycerin in mice, we found tolerance developed before 2 days,^18^ whereas G1 continued to work at 4 days, which was the longest duration examined. Any future studies might also investigate whether G1, or indeed any optimized analogues, work as an antihypertensive in other models of hypertension.

With the considerations above in mind, we set out to identify compounds that induce the oxidative activation of PKG Iα, with subsequent studies to further assess candidates identified for their ability to dilate resistance blood vessels and lower blood pressure in vivo. Our vision was that drug-like molecules capable of inducing or mimicking disulfide of PKG Iα would have electrophilic properties. Consequently, we assembled a small chemical library (Table S1), deliberately selecting compounds with features that are anticipated to result in reactivity with thiolates. Such compounds with potential for protein conjugation are normally excluded from drug libraries as they have been thought to have significant potential for nonselective, broad, and irreversible modification of proteins. This historical view of so-called covalent drugs that adduct proteins is changing, with the realization that such compounds may offer selectivity, potency, and pharmacodynamic advantages over traditional drugs that bind targets reversibly.^21–23^ Furthermore, it should be remembered that commonly used drugs such as aspirin, penicillin, and omeprazole mediate their actions via covalent adduction mechanisms.^24^

To avoid widespread modification of disparate thiols, an electrophilic drug is likely to have specific features that enable this. The thiol-reactive electrophilic moiety would likely have relatively low reactivity with cysteines to avoid rapid reaction with abundant protein or nonprotein thiols such as glutathione. In this regard, despite its abundance, reduced glutathione is not a major issue because the high acid dissociation constant (pKa) of its thiol renders it significantly unionized and so unreactive at physiological pH. Indeed, the same is true for the majority of protein thiols, with a key strategy in the design of covalent inhibitors being the targeting of a specific low pKa nucleophilic cysteine thiol that are absent or rare in related proteins, so limiting off-target effects in family members.^21^ An issue with limiting the electrophilicity and so reactivity of a drug is that it will not typically associate with a target protein with reactive ionised thiol (termed a thiolate) for sufficiently long to allow a reaction and so conjugation to occur. This may be overcome during the design of a covalent drug by including additional chemical features, in addition to its electrophilicity, that enable the drug to first bind to a target protein—directing it to the protein with the target thiolate. This increases the residence time of the drug with the target, allowing sufficient time for the conjugative addition reaction to occur. Once the covalent adduction occurs, it may be irreversible or only removed slowly, potentially resulting in high potency and advantageous pharmacodynamics.^21–23^ In this connection, it is notable that G1 contains an indole ring that resembles the purine ring in authentic cGMP. Even partial affinity of G1 for PKG may afford selectivity by allowing the drug and kinase to associate, providing the opportunity when is debinds for it to interact with C42 to afford oxidative activation. As other isoforms of PKG lack C42, meaning G1 cannot react with those kinases even if they transiently interact. Thus, this 2-component mechanism in which a selectivity filter is combined with soft electrophile reaction chemistry manifests as a potent and selective hit compound. This likely explains the ability of G1 to efficiently lower the blood pressure of WT, but not C42S PKG Iα KI, mice that are hypertensive. Although G1 lowered blood pressure in hypertensive animals, it failed to in those that were healthy and normotensive. However, G1 did increase heart rate in those mice, which is consistent with the anticipated reflex tachycardia in response to acute treatment with vasodilator pharmacotherapy.^15^

Attempts have been made to generate antihypertensives through the development of drugs that inhibit phosphodiesterase 5, with the anticipation that it would elevate cGMP and activate PKG to lower blood pressure. It is notable that despite the successful generation of such inhibitors, that they are rather ineffective systemic arterial vasodilators and are not used as antihypertensives.^25,26^ NO-cGMP also has complex direct effects on myocardial function with overstimulation of this pathway being negatively inotropic,^27,28^ with dysregulated excitation–contraction coupling perhaps contributing to diastolic dysfunction.^29^ In this connection, it is notable that phospholamban S16 is selectively phosphorylated by disulfide PKG Iα, to directly regulate and enhance myocardial relaxation during diastole.^30^ An implication is that G1 may offer a therapy against diastolic dysfunction, which warrants further exploration. Phosphodiesterase 5 inhibition, which can elevate cGMP, has, however, proven unsuccessful in the treatment of heart failure with preserved ejection fraction,^31^ whereas this pharmacotherapy protected against cardiac injury after transverse aortic constriction^32^ or doxorubicin chemotherapy.^33^

Although phosphodiesterase 5 inhibitors have proven rather ineffective in therapy of systemic arterial hypertension, such compounds are effective in vasodilating blood vessels from the pulmonary or penile circulation^34,35^ and consequently are effective in the treatment of pulmonary hypertension or erectile dysfunction. These observations are in line with the NO-cGMP pathway not being a major mediator of blood pressure, consistent with studies showing that EDHF mechanisms are likely more important in this regard.^6–11^ This is intriguing as clearly the NO-cGMP pathway can be recruited in the arterial system to lower blood pressure, as coadministration of the NO donor nitroglycerin with a phosphodiesterase 5 inhibitor lowers blood pressure substantively to induce hypotension, such that this dual treatment is clinically contraindicated.^25,26^ It would appear that while the systemic arterial system is equipped with the component enzymes of the NO-cGMP-PKG pathway, that this is not a major mechanism of endogenously controlled vasodilation. Perhaps, the most likely explanation for this is that the systemic resistance arteries do not generate NO, but instead, their NO synthase enzymes are uncoupled and so generate oxidants.^11^ These oxidants mediate, at least in part, the EDHF-dependent vasodilation that predominates in such resistance vessel. The drug G1 we have identified recruits the oxidative activation of PKG Iα to lower blood pressure and, as such, harnesses the major mechanism of endogenous systemic arterial vasodilation. Thus, although drugs that elevate cGMP have not proven effective as antihypertensives, compounds such as G1 that engage the oxidative activation PKG Iα may be more successful.

PKG Iα is activated by cGMP or oxidants, and both of these agents induce vasorelaxation, and so it is perhaps logical to assume that this dilatory outcome is mediated by the phosphorylation of the same substrates. This is not necessarily the case. The interprotein C42 disulfide in PKG Iα occurs within the substrate-targeting domain,^36^ and this may cause disparate targeting compared with cGMP. Vasodilation induced by oxidants is deficient in the C42S PKG KI mouse, whereas NO-induced or 8-Br-cGMP–induced vasodilation is identical in both genotypes.^12^ Essentially, the mechanism of oxidant-dependent versus NO-cGMP–dependent vasodilation is different, despite PKG Iα being involved in both of them. Consistent with this, although G1-dependent vasodilation was deficient in KI mesenteries compared with WT, there was no difference between genotypes in vasorelaxation to the cGMP-elevating agent cinaciguat in the studies reported here. 8-Br-cGMP stimulated VASP phosphorylation in smooth muscle cells, whereas administration of G1 alone did not do this. However, it was evident that G1 synergized with 8-Br-cGMP to potentiate VASP phosphorylation. It is possible that G1-induced oxidation of PKG Iα targets it to its substrates, with the 8-Br-cGMP stimulating activity of the kinase. Such a mechanism would be consistent with potentiated phosphorylation of VASP observed when both compounds were coadministered.

It is evident that G1 can have some off-target effects independently from C42 PKG Iα, as mesenteries isolated from C42S PKG Iα KI mice relaxed when higher concentrations of the compound were used. Furthermore, G1 administered by intraperitoneal injection partially reduced the blood pressure of hypertensive KI mice, although markedly less so than in hypertensive WTs. We conclude that G1 can couple to vasodilation and blood pressure lowering by a C42 PKG Iα-independent mechanism. However, it is important to highlight that when the drug was administered orally at 20 mg/kg to hypertensive mice, the compound only lowered blood pressure in WT but not KI. Because drugs are used at progressively higher concentrations, they will bind increasingly more targets and may affect the function of some of them—generating off-target effects. This may be because G1 is bioavailable at a higher concentration with intraperitoneal injection, whereas with oral administration, the drug likely does not reach the same tissue concentration, perhaps because of first-pass metabolism and inefficiencies in absorption.

## Perspectives

In summary, we have provided proof-of-principle that oxidant-induced activation of PKG Iα can be harnessed as a pharmacotherapy for the treatment of hypertension. Indeed, we have identified a hit compound, which we have called G1, which was able to efficiently lower blood pressure in hypertensive WT mice. Its inability to lower blood pressure in hypertensive C42S PKG Iα KI mice provides robust evidence for target validation. KI mouse only differs from PKG Iα in the WT mouse by a single atom,^12^ meaning that we can confidently ascribe the action of G1 to its interaction with C42 of the kinase. This hit compound could be developed into a lead, with the prospect of enhancing its potency and other desired attributes of a novel antihypertensive drug. Such improved variants of G1 could be examined more extensively in terms of their ability to lower blood pressure in a variety of models of hypertension in multiple species, perhaps including humans.

## Sources of Funding

This work was supported by the British Heart Foundation, the European Research Council (ERC Advanced award), the Medical Research Council, and the Department of Health via the NIHR cBRC award to Guy’s & St Thomas’ NHS Foundation Trust. P. Eaton is supported by a Grants4Targets award from Bayer Pharma AG that relates to drug-induced oxidative activation of PKG Iα.

## Disclosures

None.

## Supplementary Material

## Novelty and Significance

### What Is New?

- We have provided proof-of-principle for a new class of antihypertensive drugs.
- The hit compound we have identified induces vasodilation and blood pressure lowering via the oxidative activation of PKG Iα (protein kinase G Iα).

### What Is Relevant?

- These observations are relevant in vivo as C42S PKG Iα knockin mice, which express an engineered form of the kinase that cannot be activated by oxidants, were resistant to the compound we have identified.
- The hit compound we have identified harnesses an endogenous vasodilatory mechanism not used by current pharmacotherapies and as such may provide an effective complementary strategy for combating high blood pressure or perhaps resistant hypertension.

### Summary

The oxidative activation of PKG Iα is a significant endogenous mechanism of vasodilation and blood pressure-lowering that is not harnessed by current antihypertensive pharmacotherapies. We have identified a hit compound that recruits this mechanism and it has proved efficient in lowering blood pressure in vivo in a model of murine hypertension. Our observations provide proof-of-principle for a new class of antihypertensive drugs that have potential for further development for clinical use in humans.
