# Supplementary material for: Proof of Principle for a Novel Class of Antihypertensives That Target the Oxidative Activation of PKG Iα (Protein Kinase G Iα)
Source: Hypertension. 2017 Aug 9;70(3):577–86. doi: 10.1161/HYPERTENSIONAHA.117.09670 (PMC5548503; doi:10.1161/HYPERTENSIONAHA.117.09670)
Supplement: Supplementary file 1 [file hyp-70-577-s001.pdf]

ONLINE SUPPLEMENT

Proof-of-principal for a novel class of anti-hypertensives that target the oxidative activation of Protein Kinase G Iα

Joseph R. Burgoyne, Oleksandra Prysyazhna, Daniel A. Richards and Philip Eaton.

King's College London, Cardiovascular Division, the British Heart Foundation Centre of Excellence, the Rayne Institute, St Thomas' Hospital, London, SE1 7EH, UK.

Address for correspondence:

Joseph Burgoyne, Philip Eaton

King's College London,

Cardiovascular Division,

the Rayne Institute, St Thomas' Hospital,

London, SE1 7EH, UK.

Telephone: +44(0)2021880969

Fax: +44(0)2021880970

E-mail: joseph.burgoyne@kcl.ac.uk

E-mail: philip.eaton@kcl.ac.uk

Running title: towards a novel class of anti-hypertensives

**Table S1**			
**Molecules from Interbioscreen used in this study**			
		Index	
1	STOCK1N-03030	DNC	
2	STOCK1N-19321	DNC	
3	STOCK1N-51902	DNC	
4	STOCK1N-06800	DNC	
5	STOCK1N-31035	RAR	
6	STOCK1N-08486	RAR	
7	STOCK1N-05758	RAR	
8	STOCK1N-07458	RAR	
9	STOCK1N-03196	RAR	
10	STOCK1N-57611	RAR	
11	STOCK1N-31011	DNC	
12	STOCK1N-73661	DNC	
13	STOCK1N-08621	RAR	
14	STOCK1N-06310	DNC	
15	STOCK1N-06691	RAR	
16	STOCK1N-04694	RAR	
17	STOCK1N-11714	RAR	
18	STOCK1N-03735	RAR	
19	STOCK1N-55511	DNC	
20	STOCK1N-16313	RAR	
21	STOCK1N-06029	DNC	
22	STOCK1N-54912	DNC	
23	STOCK1N-57526	DNC	
24	STOCK1N-58013	DNC	
25	STOCK1N-05620	DNC	
26	STOCK1N-56155	DNC	
27	STOCK1N-24711	DNC	
28	STOCK1N-71851	GNC	
29	STOCK1N-49547	GNC	
30	STOCK1N-09101	GNC	
31	STOCK1N-54517	GNC	
32	STOCK1N-09726	RAR	
33	STOCK1N-46228	DNC	
34	STOCK1N-44427	GNC	
35	STOCK1N-16691	DNC	
36	STOCK1N-13224	DNC	
37	STOCK1N-06594	DNC	
38	STOCK1N-09840	DNC	
39	STOCK1N-24416	DNC	
40	STOCK1N-01339	DNC	
41	STOCK1N-01103	DNC	
42	STOCK1N-14204	DNC	
43	STOCK1N-12472	DNC	
44	STOCK1N-03079	RAR	
45	STOCK1N-11306	DNC	

Molecules were obtained from Interbioscreen
(http://www.interbioscreen.com)

GNC	Rare derivatives
DNC	
RAR	

highlighted molecules indicate those investigated in greater detail in this study

46	STOCK1N-43894	DNC						
47	STOCK1N-45388	DNC						
48	STOCK1N-05887	DNC						
49	STOCK1N-05728	DNC						
50	STOCK1N-06289	DNC						
51	STOCK1N-11489	DNC						
52	STOCK1N-51795	DNC						
53	STOCK1N-04305	DNC						
54	STOCK1N-03280	DNC						
55	STOCK1N-11383	DNC						
56	STOCK1N-11268	DNC						
57	STOCK1N-03166	DNC						
58	STOCK1N-57050	RAR						
59	STOCK1N-57713	RAR						
60	STOCK1N-30541	RAR						
61	STOCK1N-73226	DNC						
62	STOCK1N-18635	DNC						
63	STOCK1N-07999	RAR						
64	STOCK1N-10126	DNC						
65	STOCK1N-06505	DNC						
66	STOCK1N-67880	GNC						
67	STOCK1N-02870	DNC						
68	STOCK1N-57870	DNC						
69	STOCK1N-28013	DNC						
70	STOCK1N-24598	DNC						
71	STOCK1N-44554	DNC						
72	STOCK1N-06387	DNC						
73	STOCK1N-05437	RAR						
74	STOCK1N-08900	RAR						
75	STOCK1N-06813	RAR						
76	STOCK1N-00851	RAR						
77	STOCK1N-06530	RAR						
78	STOCK1N-04678	DNC						
79	STOCK1N-09835	RAR						
80	STOCK1N-07034	RAR						
81	STOCK1N-57116	RAR						
82	STOCK1N-23279	DNC						
83	STOCK1N-52322	GNC						
84	STOCK1N-27040	DNC						
85	STOCK1N-30214	DNC						
86	STOCK1N-46720	RAR						
87	STOCK1N-43337	RAR						
88	STOCK1N-55604	RAR						
89	STOCK1N-42722	RAR						
90	STOCK1N-24462	RAR						
91	STOCK1N-16139	RAR						
92	STOCK1N-15902	RAR						
93	STOCK1N-23698	RAR						

94	STOCK1N-22989	RAR						
95	STOCK1N-24428	RAR						
96	STOCK1N-16066	RAR						
97	STOCK1N-16133	RAR						
98	STOCK1N-16074	RAR						
99	STOCK1N-24719	RAR						
100	STOCK1N-24143	RAR						
101	STOCK1N-22983	RAR						
102	STOCK1N-15978	RAR						
103	STOCK1N-16045	RAR						
104	STOCK1N-15956	RAR						
105	STOCK1N-60636	RAR						
106	STOCK1N-20153	RAR						
107	STOCK1N-18419	RAR						
108	STOCK1N-18346	RAR						
109	STOCK1N-18146	RAR						
110	STOCK1N-52556	RAR						
111	STOCK1N-19596	RAR						
112	STOCK1N-18644	RAR						
113	STOCK1N-21191	RAR						
114	STOCK1N-19150	RAR						
115	STOCK1N-20633	RAR						
116	STOCK1N-18712	RAR						
117	STOCK1N-19966	RAR						
118	STOCK1N-43066	RAR						
119	STOCK1N-46335	RAR						
120	STOCK1N-70189	RAR						
121	STOCK1N-45354	RAR						
122	STOCK1N-41727	RAR						
123	STOCK1N-44160	RAR						
124	STOCK1N-47164	RAR						
125	STOCK1N-42617	GNC						
126	STOCK1N-68145	GNC						
127	STOCK1N-54500	GNC						
128	STOCK1N-67126	GNC						
129	STOCK1N-71298	DNC						
130	STOCK1N-31311	GNC						
131	STOCK1N-52625	GNC						
132	STOCK1N-53959	GNC						
133	STOCK1N-03926	GNC						
134	STOCK1N-69096	GNC						
135	STOCK1N-54241	DNC						
136	STOCK1N-23407	GNC						
137	STOCK1N-24052	RAR						
138	STOCK1N-23387	RAR						
139	STOCK1N-29232	RAR						
140	STOCK1N-28930	RAR						
141	STOCK1N-24697	RAR						

142	STOCK1N-23553	RAR						
143	STOCK1N-24227	RAR						
144	STOCK1N-23949	RAR						
145	STOCK1N-22652	RAR						
146	STOCK1N-23355	RAR						
147	STOCK1N-22664	RAR						
148	STOCK1N-23842	RAR						
149	STOCK1N-23931	RAR						
150	STOCK1N-22702	RAR						
151	STOCK1N-24439	RAR						
152	STOCK1N-23040	RAR						
153	STOCK1N-29531	RAR						
154	STOCK1N-23573	RAR						
155	STOCK1N-23677	RAR						
156	STOCK1N-23484	RAR						
157	STOCK1N-29279	RAR						
158	STOCK1N-28434	RAR						
159	STOCK1N-24233	RAR						
160	STOCK1N-29099	RAR						
161	STOCK1N-29501	RAR						
162	STOCK1N-24327	RAR						
163	STOCK1N-23542	RAR						
164	STOCK1N-22804	RAR						
165	STOCK1N-23301	RAR						
166	STOCK1N-24768	RAR						
167	STOCK1N-23662	RAR						
168	STOCK1N-24765	RAR						
169	STOCK1N-28774	RAR						
170	STOCK1N-24006	GNC						
171	STOCK1N-49811	GNC						
172	STOCK1N-03225	GNC						
173	STOCK1N-10795	RAR						
174	STOCK1N-01204	RAR						
175	STOCK1N-45544	GNC						
176	STOCK1N-07902	RAR						
177	STOCK1N-30669	RAR						
178	STOCK1N-10062	RAR						
179	STOCK1N-55476	RAR						
180	STOCK1N-08511	RAR						
181	STOCK1N-11569	RAR						
182	STOCK1N-25913	RAR						
183	STOCK1N-02920	DNC						
184	STOCK1N-00374	DNC						
185	STOCK1N-34963	GNC						
186	STOCK1N-16515	GNC						
187	STOCK1N-58824	DNC						
188	STOCK1N-69156	DNC						
189	STOCK1N-48711	RAR						

190	STOCK1N-16916	DNC						
191	STOCK1N-17002	DNC						
192	STOCK1N-69281	RAR						
193	STOCK1N-54462	RAR						
194	STOCK1N-53052	RAR						
195	STOCK1N-51383	RAR						
196	STOCK1N-50855	RAR						
197	STOCK1N-51705	RAR						
198	STOCK1N-52753	RAR						
199	STOCK1N-48731	DNC						
200	STOCK1N-48793	DNC						
201	STOCK1N-47394	DNC						
202	STOCK1N-47851	DNC						
203	STOCK1N-48774	DNC						
204	STOCK1N-48724	RAR						
205	STOCK1N-48732	DNC						
206	STOCK1N-48122	DNC						
207	STOCK1N-49123	DNC						
208	STOCK1N-48619	DNC						
209	STOCK1N-48644	DNC						
210	STOCK1N-47748	DNC						
211	STOCK1N-49178	DNC						
212	STOCK1N-47530	DNC						
213	STOCK1N-48682	RAR						
214	STOCK1N-49242	RAR						
215	STOCK1N-47727	RAR						
216	STOCK1N-48887	RAR						
217	STOCK1N-49323	RAR						
218	STOCK1N-47619	RAR						
219	STOCK1N-49446	RAR						
220	STOCK1N-48033	RAR						
221	STOCK1N-47665	RAR						
222	STOCK1N-48794	DNC						
223	STOCK1N-47453	DNC						
224	STOCK1N-49121	DNC						
225	STOCK1N-49274	DNC						
226	STOCK1N-49196	DNC						
227	STOCK1N-48985	DNC						
228	STOCK1N-49327	DNC						
229	STOCK1N-47529	DNC						
230	STOCK1N-48943	DNC						
231	STOCK1N-47786	DNC						
232	STOCK1N-48358	DNC						
233	STOCK1N-48195	DNC						
234	STOCK1N-49099	DNC						
235	STOCK1N-48164	DNC						
236	STOCK1N-47877	DNC						
237	STOCK1N-47885	DNC						

238	STOCK1N-48800	DNC						
239	STOCK1N-48684	DNC						
240	STOCK1N-47657	RAR						
241	STOCK1N-48323	DNC						
242	STOCK1N-47810	DNC						
243	STOCK1N-51495	RAR						
244	STOCK1N-56772	DNC						
245	STOCK1N-50434	DNC						
246	STOCK1N-66345	DNC						
247	STOCK1N-44321	DNC						
248	STOCK1N-69994	GNC						
249	STOCK1N-57765	DNC						
250	STOCK1N-56995	GNC						
251	STOCK1N-62919	DNC						
252	STOCK1N-64263	DNC						
253	STOCK1N-59361	RAR						
254	STOCK1N-61856	RAR						
255	STOCK1N-63076	RAR						
256	STOCK1N-59711	RAR						
257	STOCK1N-61627	RAR						
258	STOCK1N-59481	RAR						
259	STOCK1N-59372	RAR						
260	STOCK1N-63343	RAR						
261	STOCK1N-65892	DNC						
262	STOCK1N-66001	DNC						
263	STOCK1N-65962	DNC						
264	STOCK1N-65813	DNC						
265	STOCK1N-59567	RAR						
266	STOCK1N-59597	RAR						
267	STOCK1N-59760	RAR						
268	STOCK1N-62340	RAR						
269	STOCK1N-60376	RAR						
270	STOCK1N-60669	RAR						
271	STOCK1N-63584	RAR						
272	STOCK1N-60559	RAR						
273	STOCK1N-61917	RAR						
274	STOCK1N-59127	RAR						
275	STOCK1N-67951	GNC						
276	STOCK1N-06778	DNC						
277	STOCK1N-50253	GNC						
278	STOCK1N-12110	RAR						
279	STOCK1N-65687	DNC						
280	STOCK1N-62829	RAR						
281	STOCK1N-62053	RAR						
282	STOCK1N-61538	RAR						
283	STOCK1N-63309	RAR						
284	STOCK1N-59395	RAR						
285	STOCK1N-60563	RAR						

286	STOCK1N-58990	RAR						
287	STOCK1N-59303	RAR						
288	STOCK1N-61829	RAR						
